# Metabolomic and transcriptomic analyses unveil the accumulation of shikimic acid in the leaves of *Ginkgo biloba*

**DOI:** 10.3389/fpls.2025.1631197

**Published:** 2025-08-22

**Authors:** Wanwen Yu, Minyue Cai, Chenxi You, Wenxuan Wei, Huimin Liu

**Affiliations:** ^1^ National Key Laboratory for Development and Utilization of Forest Food Resources, Co-Innovation Center for Sustainable Forestry in Southern China, Nanjing Forestry University, Nanjing, China; ^2^ State Key Laboratory of Tree Genetics and Breeding, Research Institute of Non-Timber Forestry, Chinese Academy of Forestry, Zhengzhou, China

**Keywords:** shikimic acid, ginkgo, flavonoid, metabolome, transcriptome, transcription factor

## Abstract

**Introduction:**

Shikimic acid, as a critical precursor for oseltamivir synthesis in antiviral pharmaceuticals, faces escalating global demand. Although *Ginkgo biloba* leaves have emerged as a promising natural source of shikimic acid owing to their exceptional content of this valuable compound and substantial biomass production capacity, the molecular mechanisms underlying its biosynthesis and downstream metabolic regulation in *G. biloba* leaves remain largely unknown.

**Methods:**

Here, the concentration of shikimic acid in 33 clones were assessed, and 1# (referred as HS) had the highest level. The shikimic acid content in HS was 119% higher than that in 24# (referred as LS), which possessed the lowest shikimic acid level. Concurrently, we analyzed downstream metabolites including flavonoids, phenylalanine, tryptophan and tyrosine, along with transcriptomic and metabolomic profiles in HS and LS.

**Results:**

The concentrations of flavonoids, phenylalanine, tryptophan and tyrosine in HS were markedly lower than those in LS. Principal component analysis (PCA) and partial least squares discriminant analysis (PLS-DA) analyses revealed clear differences in metabolites between HS and LS. Numerous metabolites and genes related to biosynthesis and downstream metabolic partitioning of shikimic acid were significantly differentially regulated. For instance, the transcript levels of *malate dehydrogenase* (*MDH*) and *ribose-5-phosphate isomerase* (*RPI*), that are involved in shikimic acid biosynthesis, were more upregulated in HS compared to LS. The abundances of tyrosine, tryptophan, luteolin and dihydromyricetin and the mRNA levels of *chorismate synthase* (*CS*), *chalcone synthase* (*CHS*), *chalcone isomerase* (*CHI*) and *flavanone-3b-hydroxylase* (*F3H*), that are implicated in downstream metabolism of shikimic acid were downregulated in HS compared to LS. Additionally, the abundances of abscisic acid and auxin in HS were lower than those in LS. Through association analysis, 27 metabolites, 33 structural genes and 28 transcription factors, such as *ERFs*, *C2H2s* and *MYBs* that play roles in shikimic acid accumulation were identified.

**Conclusion:**

These results suggest that metabolites and structural genes participating in biosynthesis and downstream metabolism of shikimic acids, and phytohormones and transcript factors play essential roles in shikimic acid accumulation in *G. biloba* leaves.

## 1 Introduction

Shikimic acid (3, 4, 5-trihydroxy cyclohexene carboxylic acid), as a natural occurring hydroaromatic compound with chiral characteristics (Quan et al., 2012), has antioxidant, anti-inflammatory and antiviral activities (Marchiosi et al., 2019; Gandhi et al., 2023). Shikimic acid is generally utilized as an essential precursor for synthesizing Oseltamivir, which is a frontline antiviral medicine critical for prophylaxis and treatment of influenza A/B viruses (Kancharla et al., 2009; Tripathi et al., 2014). Beyond pharmaceutical applications, shikimic acid and its derivatives have substantial agricultural utility, functioning as plant growth enhancers (Al-Amri, 2013), eco-friendly herbicides and antimicrobial agents (Díaz-Quiroz et al., 2018). Despite recent advances in the processes of chemical and microbial synthesis as viable alternatives (Rawat et al., 2013; Bilal et al., 2018; Candeias et al., 2018), plant-derived shikimic acid continues to dominate industrial-scale antiviral production due to its inherent non-toxic profile (Marchiosi et al., 2019).

Shikimic acid serves as a central metabolic node within the shikimate pathway, which is a universal biosynthetic route responsible for the synthesis of flavonoids and amino acids (Kougan et al., 2013). The study about accumulation of shikimic acid has been mainly performed in bacteria, involving multiple enzymatic reactions (Wu et al., 2022; Shende et al., 2024). Briefly, the initial reaction of the shikimate synthetic pathway occurs by the formation of 3-deoxy-D-arabinoheptose-7-phosphate (DAHP) via condensation of phosphoenolpyruvate (PEP) and erythrose-4-phosphate (E4P) (Herrmann and Weaver, 1999). Subsequently, 3-dehydroquinate (DHQ) synthase catalyzes the conversion of DAHP into DHQ, which is further dehydrated by DHQ dehydrase into 3-dehydroshikimate (DHS) (Maeda and Dudareva, 2012). NADPH-dependent shikimate dehydrogenase then catalyzes the reduction of DHS to yield shikimic acid. Thereafter, shikimate kinase (SK) catalyzes the production of shikimate-3-phosphate (S3P) (Tzin et al., 2012). S3P is subsequently converted to 5-enolpyruvylshikimate-3-phosphate (EPSP) under the catalyzes of EPSP synthase (Wang et al., 2017; Gandhi et al., 2023). Chorismate synthase (CS) ultimately transforms EPSP into chorismate, which is a pivotal branch-point metabolite that feeds into the biosynthesis of aromatic amino acids (phenylalanine, tryptophan and tyrosine) and other secondary metabolites (Gu et al., 2017). Despite its pharmacological significance, critical knowledge gaps persist regarding the regulatory mechanisms governing shikimic acid accumulation in plants.

The current industrial production of shikimic acid relies on extraction from mature fruits of Chinese star anise (*Illicium verum*). Thus, the fruiting season and production of mature fruits limits the output of shikimic acid. Recent phytochemical analysis by Kulić et al. (2022) found that the concentration of shikimic acid in *Ginkgo biloba* leaves is about 20 mg g^-1^, which is lower than the 66 mg g^-1^ reported in mature fruits of Chinese star anise (Ramazani et al., 2021). However, the annual biomass yield of *G. biloba* leaves is much higher than mature fruits of Chinese star anise. Moreover, given their renewable nature and rapid harvest cycles, *G. biloba* leaves present a promising alternative source for this pharmacologically significant precursor compound. Other plant species, such as sweetgum (*Liquidambar styraciflua*) and *Pinus elliottii*, have also been reported to produce shikimic acid (Martin et al., 2010; Xie et al., 2012). However, sweetgum-derived shikimic acid is extracted from non-renewable bark and wood tissues, while *P. elliottii* needles contain lower concentrations compared to *G. biloba* leaves (Xie et al., 2012). Although microbial synthesis using genetically engineered *Escherichia coli* has become an important alternative approach (Bilal et al., 2018), this method often relies on costly substrates. In contrast, *G. biloba* is widely cultivated and generates substantial leaf biomass annually, making it an ecologically sustainable and economically feasible plant-based source.

As a gymnosperm species belonging to Ginkgopsida, *G. biloba* L. is extensively cultivated in China, Korea, and Japan (Zhao et al., 2010; Crane, 2018). Its leaves extracts have various health benefits, such as anti-inflammatory, neuroprotective and anti-aging properties (Yu et al., 2022). Additionally, in order to preserve old books, *G. biloba* leaves are used to be inserted among the pages, demonstrating *G. biloba* leaves play an important role in traditional Chinese culture. Nevertheless, despite generating substantial biomass in Chinese plantations, current utilization remains insufficient, and only 1.5–2.9% of the leaf biomass ends up with a valuable product (Kulić et al., 2023). The extraction of shikimic acid from *G. biloba* leaves not only holds significant economic value but also promotes sustainable resource management. Noticeably, our preliminary experiments have shown that the content of shikimic acid varies greatly among different *G. biloba* clones. Similarly, different ginkgo clones also exhibit contrasting flavonoid concentration in the leaves. For instance, Yao et al. (2012) found that the flavonol glycoside content in Anjie (a ginkgo clone) was 19.19 times higher than that in TaiXing (a ginkgo clone), which possessed the lowest level. It is of great significance to screen *G. biloba* clones with great shikimic acid concentration, and further analyze the molecular mechanism underlying shikimic acid accumulation in *G. biloba* leaves.

Here, the leaves from 33 *G. biloba* clones were collected, respectively, and the content of shikimic acid was quantified. Metabolome and transcriptome were employed to detect the abundances of metabolites and expression levels of genes in the leaves of *G. biloba*. The aims of this study were to (i) identify *G. biloba* clones with high shikimic acid content, (ii) identify metabolites, structural genes and transcript factors related to shikimic acid accumulation in *G. biloba* leaves.

## 2 Materials and methods

### 2.1 Plant materials and harvest

From the *G. biloba* nursery in Nanjing Forestry University (32° 04’N, 118° 48’E, Nanjing, China), 33 clones (namely 1#-33#, 18-year-old) with leaf utilization potential were selected. These clones were originated from Pizhou, Jiangsu Province, and were selected in 2005 based on seedling height and ground diameter. Afterward, these clones were transplanted into a common garden in Nanjing forestry university with uniform spacing (4m × 4m) to minimize environmental variation. In mid-April, the healthy functional leaves were harvested from every clone and immediately wrapped in tinfoil and frozen in liquid nitrogen. Leaf samples were ground into fine powder with a mortar and a pestle in liquid nitrogen. The fine-powdered samples were then stored at an ultralow temperature refrigerator for further analyses. For each clone, six branches that exhibited similar growth status and exposed to sunlight were selected. For physiological and metabolomic analyses, leaves from each branch were collected separately to form an individual sample, resulting in six biological replicates for each clone. For the transcriptomic analysis, equal amounts of leaf samples from every two branches were pooled together to form a mixed sample. Consequently, three mixed samples were obtained for each clone. 

### 2.2 Determination of shikimic acid

The concentration of shikimic acid was measured as described by Zelaya et al. (2011) and Joubert et al. (2023) with minor modifications. Briefly, ca. 50 mg oven-dried fine powders were mixed with 0.25 mol L^-1^ hydrochloric acid for 30 min. After centrifuged (6000 g) for 10 min, the supernatant was collected and diluted three times. The reaction was initiated by adding 1% (w/v) periodate solution. Three hours later, the reaction was stopped by adding a solution containing 1 mol L^-1^ NaOH and 1mol L^-1^ glycinate. Shikimic acid concentration was measured spectrophotometrically at 380 nm.

### 2.3 Determination of phenylalanine, tryptophan and tyrosine

Phenylalanine concentration was assayed using a phenylalanine content kit (Nanjing Jiancheng Bioengineering Research Institute Co., Nanjing, China) following the kit’s instructions.

Tryptophan and tyrosine were assayed as described previously (Park et al., 2012; Botella et al., 2023) with minor modifications. Briefly, frozen powder was homogenized in 100 µL extraction solution containing 50% ethanol and 0.1 mol L^-1^ HCl. The mixture was centrifuged (13800 g, 4°C, 20 min), and the supernatant was filtered through an organic membrane (0.22 μm). The filtered supernatant was used to determine tryptophan and tyrosine with liquid chromatography-mass spectrometry (LC/MS, LTQ-XL, Thermo Scientific, Waltham, MA, USA).

### 2.4 Determination of flavonoid

The concentration of flavonoid in the leaves were analyzed as described previously (An et al., 2024; Cui et al., 2025). Briefly, oven-dried fine powder (ca. 200 mg) was packed with filter paper and washed with 100 mL petroleum ether in Soxhlet extraction to remove impurities for 8 h. Afterward, the purified packed fine powder was extracted in 10 mL methanol at 60 °C for 30 min, and this procedure was repeated twice. The extraction was transferred into a new volumetric flask, ensuring the final volume to 20 mL. The extraction was homogenized with 5% sodium nitrite, 10% aluminum nitrate, and 1 mol L^-1^ NaOH for 5, 6 and 10 min, respectively. The absorbance of the mixture was determined spectrophotometrically at 510 nm.

### 2.5 Metabolite profiling analysis

**Figure 1:**
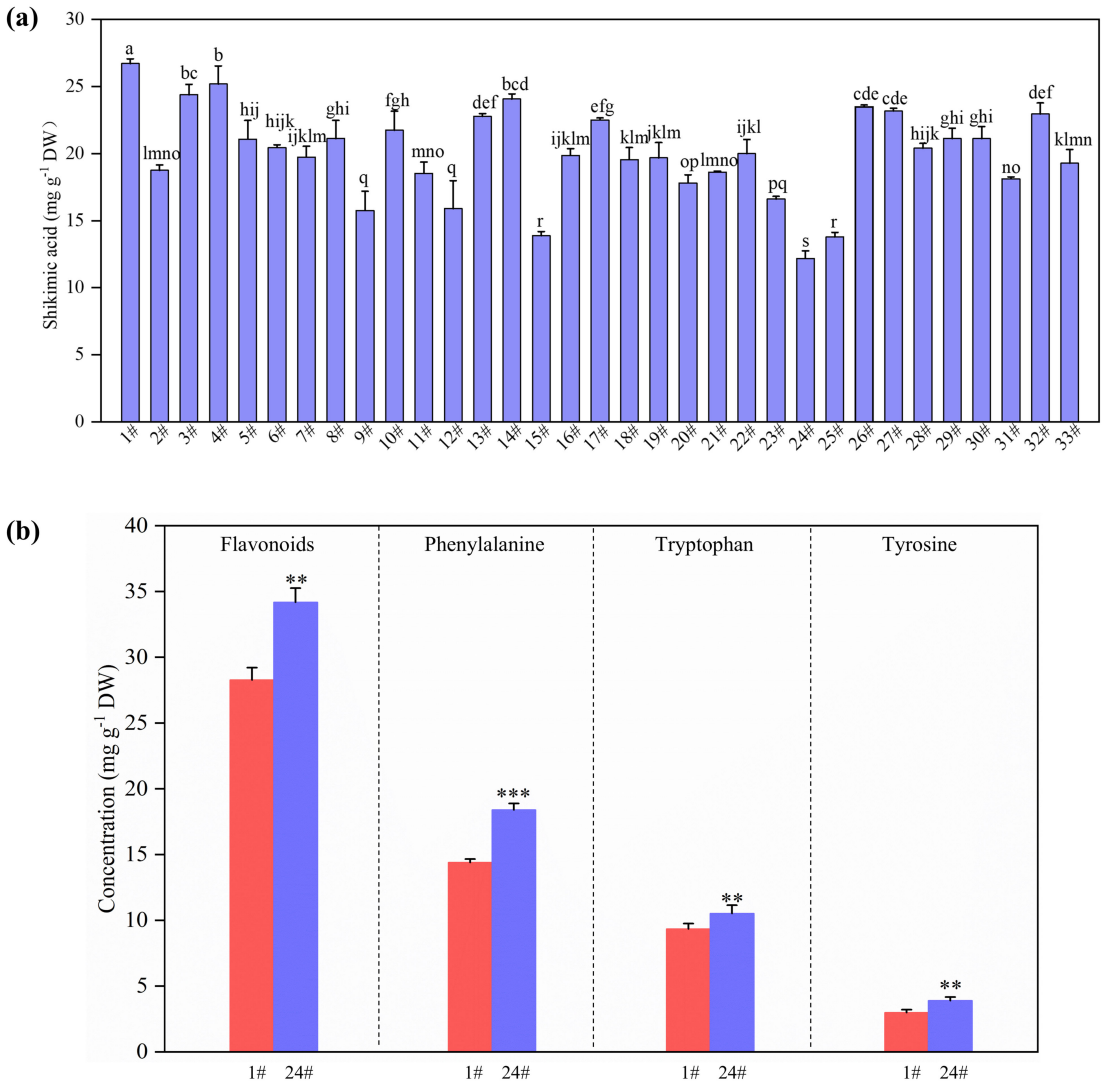
Concentration of shikimic acid in the leaves of 33 *Ginkgo biloba* clones **(a)**, and concentrations of flavonoids, phenylalanine, tryptophan and tyrosine in the leaves of 1# and 24# **(b)**. *P*-values obtained from one-way ANOVA test are indicated: *P < 0.05; **P < 0.01; ***P < 0.001.

Since shikimic acid content in 1# (referred as HS) was the highest while that in 24# (referred as LS) was the lowest (**Figure 1a**), 1# and 24# were selected to explore metabolomic analysis of shikimic acid synthesis and downstream metabolism. The clones of No.1 (HS) and No.24 (LS) originate from distinct individual trees and are not derived from mutated shoots of any single tree. Nontargeted metabolites in the leaves were analyzed based on the method described previously (Yu et al., 2022; Hong et al., 2023). Briefly, fresh fine powders (ca. 100 mg) were extracted with 500 μL of 80% methanol and were incubated on ice for 5 min. The mixture was centrifuged (15000 g, 4°C) for 20 min. The collected supernatant was diluted with mass spectrometry water until methanol content was 53%. Then, the samples were transferred to fresh tubes and centrifuged (15000 g, 4°C) for 20 min. Finally, the supernatant was collected and injected into a Vanquish UHPLC system (Thermo Fisher Scientific, Waltham, MA, USA) coupled with an Orbitrap Q ExactiveTM HF-X mass spectrometer (Thermo Fisher Scientific) for liquid chromatography tandem mass spectrometry (LC-MS/MS) analysis.

LC–MS/MS raw data were processed using the Compound Discoverer 3.1 (CD3.1, Thermo Fisher Scientific) to perform peak alignment, picking and area quantification. Subsequently, the precise qualitative and relative quantitative results were obtained by matching the peaks with mzCloud, mzVault and MassList databases. The metabolites were annotated using the KEGG database, HMDB database and LIPIDMaps database (Marco-Ramell et al., 2018). The metabolites with VIP > 1, *P*-value < 0.05 and log_2_ (Fold change) ≥ 1 (or ≤ -1) were considered to be differentially regulated. Notably, electrospray ionization (ESI) was employed as the ion source, and was conducted in both positive and negative ion modes.

### 2.6 RNA sequencing and bioinformatic analysis

Total RNA from the leaves of HS and LS was isolated using a polysaccharide polyphenol plant total RNA kit (DP441, TianGen, Beijing, China), respectively. RNA integrity was evaluated using Agilent 2100 bioanalyzer. Subsequently, cDNA libraries were constructed and sequenced on illumina NovaSeq 6000 (Illumina, San Diego, CA, USA). The analysis of RNA sequencing data was performed as described earlier (Lu et al., 2022; Du et al., 2024). Briefly, the original sequencing data were filtered to obtain clean data. The clean reads were then aligned to the *G. biloba* genome (Guan et al., 2016) (Genome ID: 100613, available at http://gigadb.org/dataset/100613) using HISAT2 (v.2.0.5). StringTie software was used to assemble the new transcripts, and featureCounts (v.1.5.0-p3) was used to calculate the Fragments Per Kilobase Million (FPKM) of each gene. Significantly differentially expressed genes (DEGs) were identified on the basis of |log_2_ (Fold change)| ≥ 1 and a false discovery rate (FDR) < 0.05. The clusterProfiler software (v.3.8.1) was used for gene ontology (GO) and Kyoto Encyclopedia of Genes and Genomes (KEGG) analyses (Li et al., 2024b). GO terms and KEGG pathways with *P*-values < 0.05 were considered to be significantly enriched (Xiang et al., 2024). Three cDNA libraries were generated and sequenced for HS and LS, respectively. The sequencing data were deposited to the Genome Sequence Archive (GSA, https://ngdc.cncb.ac.cn/gsa/) under Project ID CRA025492.

DEGs were annotated and functional categorized as described by Yu et al. (2021); (Lu et al., 2024) with minor modifications. Briefly, the coding sequences of DEGs were retrieved from the *G. biloba*’s genomic database. The closest homologue of a *G. biloba* gene in *Arabidopsis thaliana* was identified by searching its coding sequence against the protein sequence database of *Arabidopsis* using translated nucleotide BLAST (BLASTX). Identifiers of *Arabidopsis* genes closest to these DEGs were then submitted to MapMan (http://mapman.gabipd.org/) for functional analysis.

### 2.7 Association analysis of transcription factors and DEGs involved in shikimic acid biosynthesis and downstream metabolism

Considering the critical roles of transcription factors (TFs) in shikimic acid biosynthesis and downstream metabolism, the Pearson correlation coefficient (R) between differentially expressed TFs and shikimic acid content was calculated. Candidate TFs were defined to have an absolute R-value greater than 0.92 and a *P*-value less than 0.05. To further identify key TFs regulating the accumulation of shikimic acid, R were calculated between these candidate TFs and DEGs involved in the shikimic acid biosynthesis and downstream metabolism. Significant correlations were defined as |R| > 0.95 with *P*-value < 0.05. Correlations between key TFs and DEGs were displayed using Cytoscape (v.3.10.1).

### 2.8 Integrative analysis of metabolome and transcriptome

The differentially accumulated metabolites (DAMs) and DEGs involved in shikimic acid biosynthesis and downstream metabolism were used for the integrative analysis, and the Pearson’s correlation coefficients between them were calculated (Yu et al., 2022). A correlation was considered statistically significant if the absolute value of the |R| exceeded 0.8 with a *P*-value less than 0.05. Heatmaps were used to reveal the correlation between these DAMs and DEGs.

### 2.9 Fluorescence quantitative PCR experiment

Total RNA was extracted as mentioned above. Quantitative RT-PCR (RT-qPCR) was conducted as described previously (Zhang et al., 2023). Specific primers were designed for each DEG, and *Glyceraldehyde 3- phosphate dehydrogenase* (*GAPDH*) was selected as an internal standard (**Supplementary Table S1**).

### 2.10 Statistical analysis

Statgraphics (STN, St Louis, MO, USA) was employed to do statistical tests as described previously (Lu et al., 2019, 2023). The data was tested to explore the normality prior to the analysis. One-way analysis of variance (ANOVA) was employed, and the means were regarded to be significantly different if the *P*-value was less than 0.05 on the basis of ANOVA F-test.

## 3 Results

### 3.1 Shikimic acid, flavonoids, phenylalanine, tryptophan and tyrosine

The concentration of shikimic acid in the leaves varied greatly among the 33 *G. biloba* clones (**Figure 1a**). Notably, 1# had the highest shikimic acid level and 24# possessed the lowest content (**Figure 1a**). The shikimic acid content in HS was 119% higher than that in LS (**Figure 1a**). HS and LS were selected to further study the metabolomic and transcriptomic mechanism of shikimic acid accumulation in *G. biloba* leaves. The concentrations of flavonoids, phenylalanine, tryptophan and tyrosine, which are downstream metabolites of shikimic acid, in HS were 17%, 22%, 11% and 23% lower than those in LS, respectively (**Figure 1b**).

### 3.2 Metabolomic response

**Figure 2:**
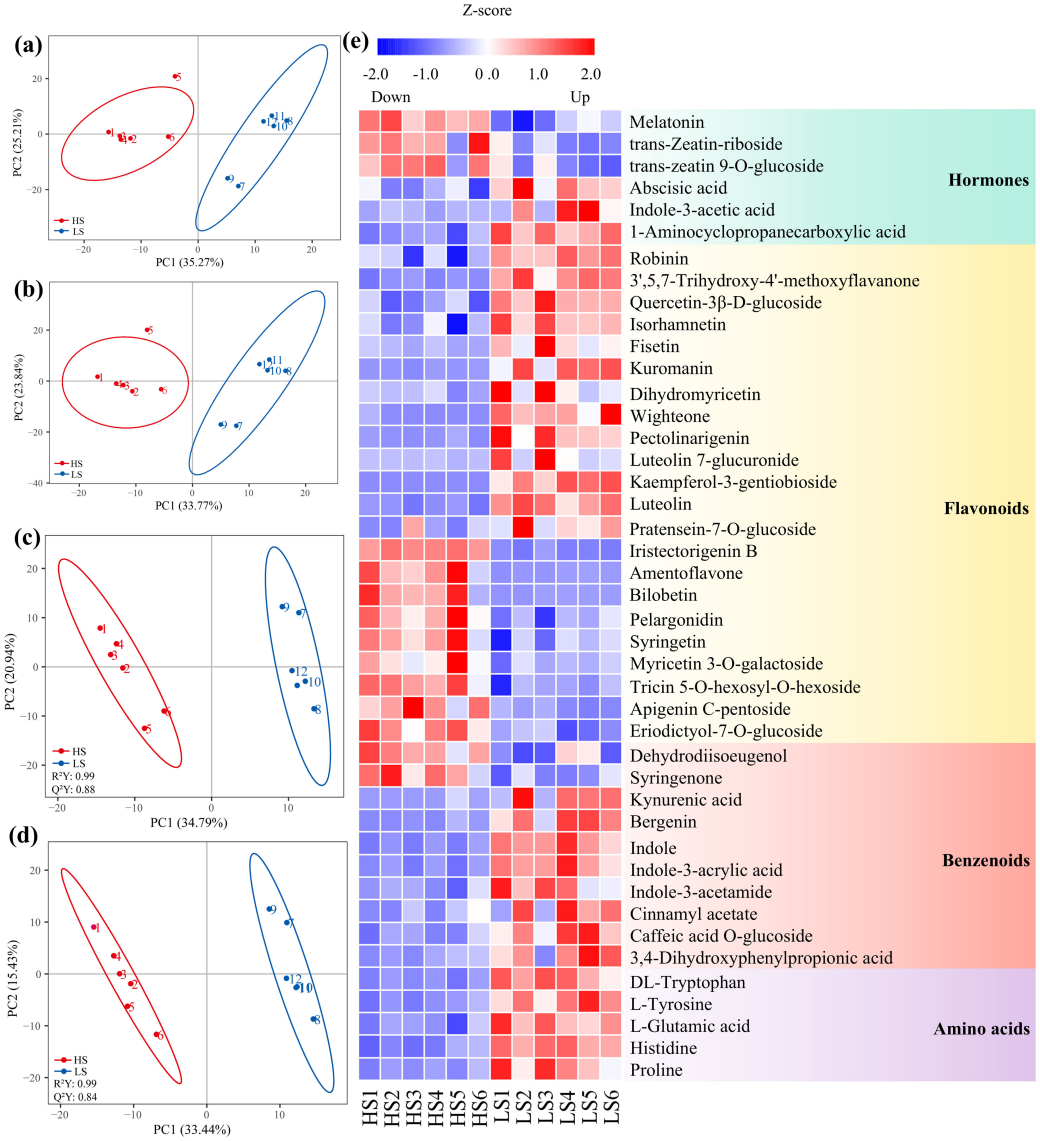
Principal component analysis (PCA) plots **(a, b)** and partial least squares discriminant analysis (PLS-DA) plots **(c, d)** of the metabolites identified in negative mode **(a, c)** and positive mode **(b, d)**, enforced with electrospray ionization (ESI) in LC-MS/MS, and hierarchical clustering of the differentially abundant metabolites between HS (1#, highest shikimic acid content) and LS (24#, lowest shikimic acid content) **(e)**. R²Y, Cumulative explained variance of the response variable in PLS-DA. Q²Y, Cross-validated predictive variance of the response variable.

A total of 862 metabolites were identified in positive (451) and negative (411) ion modes, respectively (**Supplementary Table S2**). Principal component analysis (PCA) demonstrated that the first two principal components explained 60.48% and 57.61% of the total variance in negative and positive ion modes, respectively, with PC1 serving as the dominant contributor in both analyses (**Figures 2a, b**). Partial least squares discriminant analysis (PLS-DA) showed clear metabolic differences between HS and LS (**Figures 2c, d**). In the PLS-DA model, PC1 accounted for 34.79% (negative mode) and 33.44% (positive mode), while PC2 explained 20.94% (negative mode) and 15.43% (positive mode) of variances, respectively (**Figures 2c, d**). For metabolites detected in negative ion mode, compared with LS, the abundances of 67 metabolites were higher, whereas those of 71 metabolites were lower in HS, respectively (**Supplementary Figure S1a**; **Supplementary Table S3**). 61 upregulated and 91 downregulated metabolites (in positive ion mode) were identified in HS *vs*. LS, respectively (**Supplementary Figure S1b**; **Supplementary Table S3**). According to KEGG pathway annotation, these DAMs were mainly involved in metabolic pathways, tryptophan metabolism, galactose metabolism, aminoacyl−tRNA biosynthesis, and arginine and proline metabolism (**Supplementary Figures S1c, d**).

The DAMs were divided into 10 categories, mainly including benzenoids, flavonoids, amino acids and hormones (**Figure 2e**; **Supplementary Table S3**). The abundances of most benzenoids, amino acids and hormones, were significantly lower in HS compared to those in LS (**Figure 2e**; **Supplementary Table S3**). For example, abscisic acid, indole-3-acetic acid, tyrosine, tryptophan, kynurenic acid, bergenin and 3-dehydroshikimic acid were downregulated in HS compared to LS (**Figure 2e**; **Supplementary Table S3**). The abundances of 12 flavonoid metabolites, such as luteolin, kaempferol-3-gentiobioside and wight one in HS were significantly lower when compared with those in LS (**Figure 2e**; **Supplementary Table S3**).

### 3.3 Transcriptomic response

**Figure 3:**
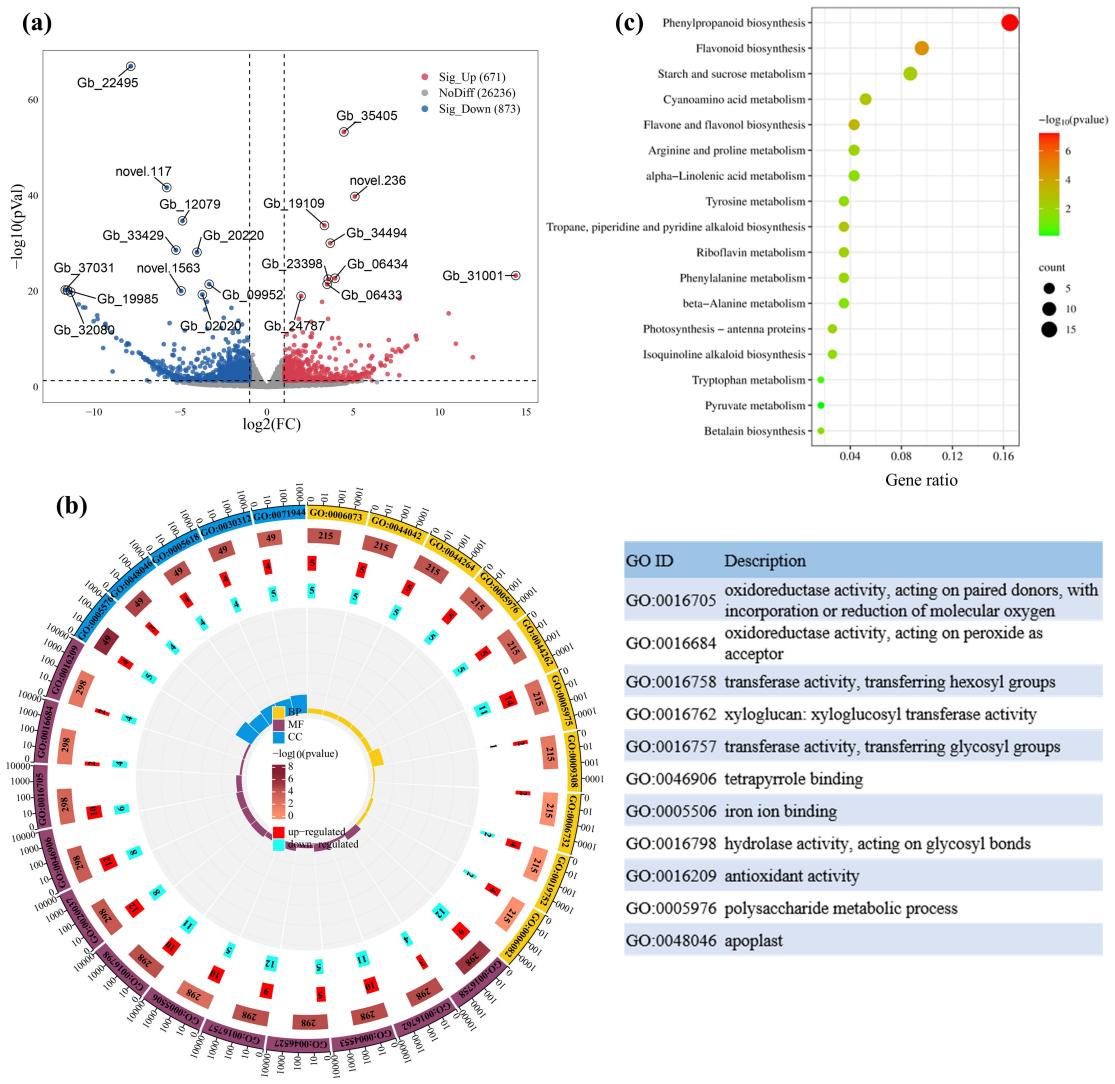
Volcano plot of significantly differentially expressed genes (DEGs) between HS and LS **(a)**, as well as gene ontology (GO) enrichment analysis **(b)**, and dot plot showing kyoto encylopaedia of genes and genomes (KEGG) enrichment analysis of DEGs **(c)**. In panel **(b)**, CC, MF, and BP represent cellular component, molecular function, and biological process, respectively. In panel **(c)**, the size of the dots represents the number of enriched genes, and the color indicates the *P*-value. Gene IDs correspond to gene models from the draft genome of *G*. *biloba* published by Guan et al. (2016) (assembly accession 100613). Detailed information about these genes is presented in **Supplementary Table S5**.

Based on the above physiological and metabolomic data, it was speculated that there are differences in gene expression patterns involved in shikimic acid synthesis and downstream metabolism between HS and LS. To test this hypothesis, the transcriptomes of leaves from HS and LS were sequenced. A total of six cDNA libraries from HS and LS were constructed, and 45.1-46.3 million clean reads were obtained from each library (**Supplementary Table S4**). 91.10-91.94% of clean reads can be mapped to the *G. biloba* genome database (**Supplementary Table S4**). Compared with LS, 671 upregulated and 873 downregulated genes were detected in HS (**Figure 3a**). Genes were randomly selected to validate the transcriptomic data using RT-qPCR (**Supplementary Figure S2**).

GO enrichment analysis showed that the differentially expressed genes (DEGs) were significantly enriched in oxidoreductase activity, acting on paired donors, with incorporation or reduction of molecular oxygen (GO:0016705), transferase activity, transferring hexosyl groups (GO:0016758), xyloglucan: xyloglucosyl transferase activity (GO:0016762), hydrolase activity, acting on glycosyl bonds (GO:0016798) and polysaccharide metabolic process (GO:0005976) (**Figure 3b**). KEGG pathway enrichment analysis showed that DEGs were involved in phenylpropanoid biosynthesis, flavonoid biosynthesis, starch and sucrose metabolism, flavone and flavonol biosynthesis, and cyanoamino acid metabolism (**Figure 3c**). These results suggest that DEGs are involved in the biosynthesis and downstream metabolism of shikimic acid. MapMan was used to further classify these DEGs into functional categories, including photosynthesis, flavonoid metabolism, hormone metabolism, amino acid metabolism and transcriptional regulation (**Supplementary Table S5**), which are closely correlated with synthesis and downstream metabolism of shikimic acid.

**Figure 4:**
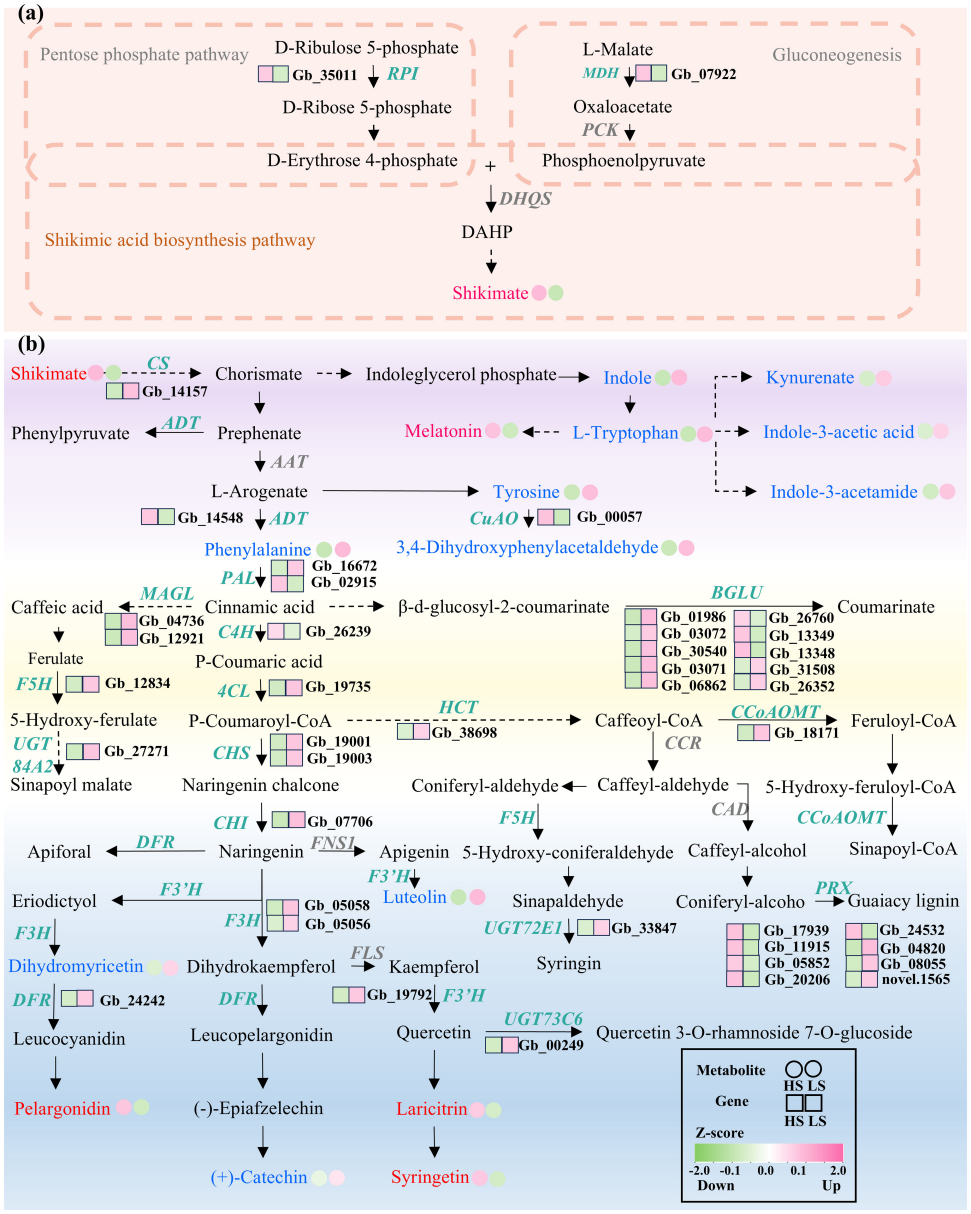
Changes in abundances of metabolites and expression levels of genes involved in shikimic acid biosynthesis **(a)** and downstream metabolism **(b)** in HS compared to LS. Metabolites marked with red/blue fonts indicate upregulated/downregulated metabolites in HS compared to LS. Genes marked with green italic fonts are significantly differentially expressed. Detailed information about these metabolites and genes is presented in **Supplementary Tables S4, S5**, respectively. Solid arrows represent single-step enzymatic reactions, while dashed arrows indicate multi-step or simplified pathways between metabolites.

In the shikimic acid synthesis pathway (**Supplementary Figure S4**), *malate dehydrogenase* (*MDH*) encoding a pivotal enzyme responsible for the conversion of l-malate into oxaloacetate (Rozova et al., 2015) and *Ribose-5-phosphate isomerase* (*RPI*) coding for a cytosolic ribose-5-phosphate isomerase that catalyzes the conversion of D-ribulose 5-phosphate to d-ribose 5-phosphate (Faria et al., 2016), were more upregulated in HS compared to LS (**Figure 4a**; **Supplementary Table S5**).

In the downstream metabolism of shikimic acid (**Supplementary Figure S4**), a number of genes that are implicated in biosynthesis of aromatic amino acids and flavonoids were downregulated in HS compared to LS (**Figure 4b**). For instance, the transcript levels of *chorismate synthase* (*CS*) encoding an enzyme that catalyzes the conversion of 5-enolpyruvylshikimate-3-phosphate (EPSP) to chorismate was downregulated in HS compared to LS (**Figure 4b**; **Supplementary Table S5**). Similarly, the mRNA levels of *4-coumarate:CoA ligase* (*4CL*) encoding an enzyme that catalyzes the ATP-dependent conversion of p-coumaric acid to p-coumaroyl-CoA, *chalcone synthase* (*CHS*) encoding a key enzyme that condenses p-coumaroyl-CoA with three molecules of malonyl-CoA to form naringenin chalcone, *flavanone-3b-hydroxylase* (*F3H*) encoding an enzyme that mediates the stereospecific 3β-hydroxylation of naringenin to dihydrokaempferol, and *dihydroflavonol 4-reductase* (*DFR*) encoding an enzyme that catalyzes the NADPH-dependent reduction of dihydromyricetin to leucocyanidin, were downregulated in HS compared to LS (**Figure 4b**; **Supplementary Table S5**).

In the category of hormone metabolism, the transcript levels of *abscisic acid-responsive element-binding factor* (*ABF*) and *gem-relate 5* (*GER5*) involved in ABA signaling (Liu et al., 2019) were downregulated in HS compared to LS (**Supplementary Figure S3**; **Supplementary Table S5**). The mRNA levels of *BIG* and *cullin-associated and neddylation dissociated* (*CAND1*) involved in auxin regulation (Gil et al., 2001; Chuang et al., 2004), and *like aux1 3* (*LAX3*) and *GH3.1* implicated in auxin transport (Kasahara et al., 2020), were downregulated in HS compared to LS (**Supplementary Figure S3**).

### 3.4 Differentially expressed transcription factors

**Figure 5:**
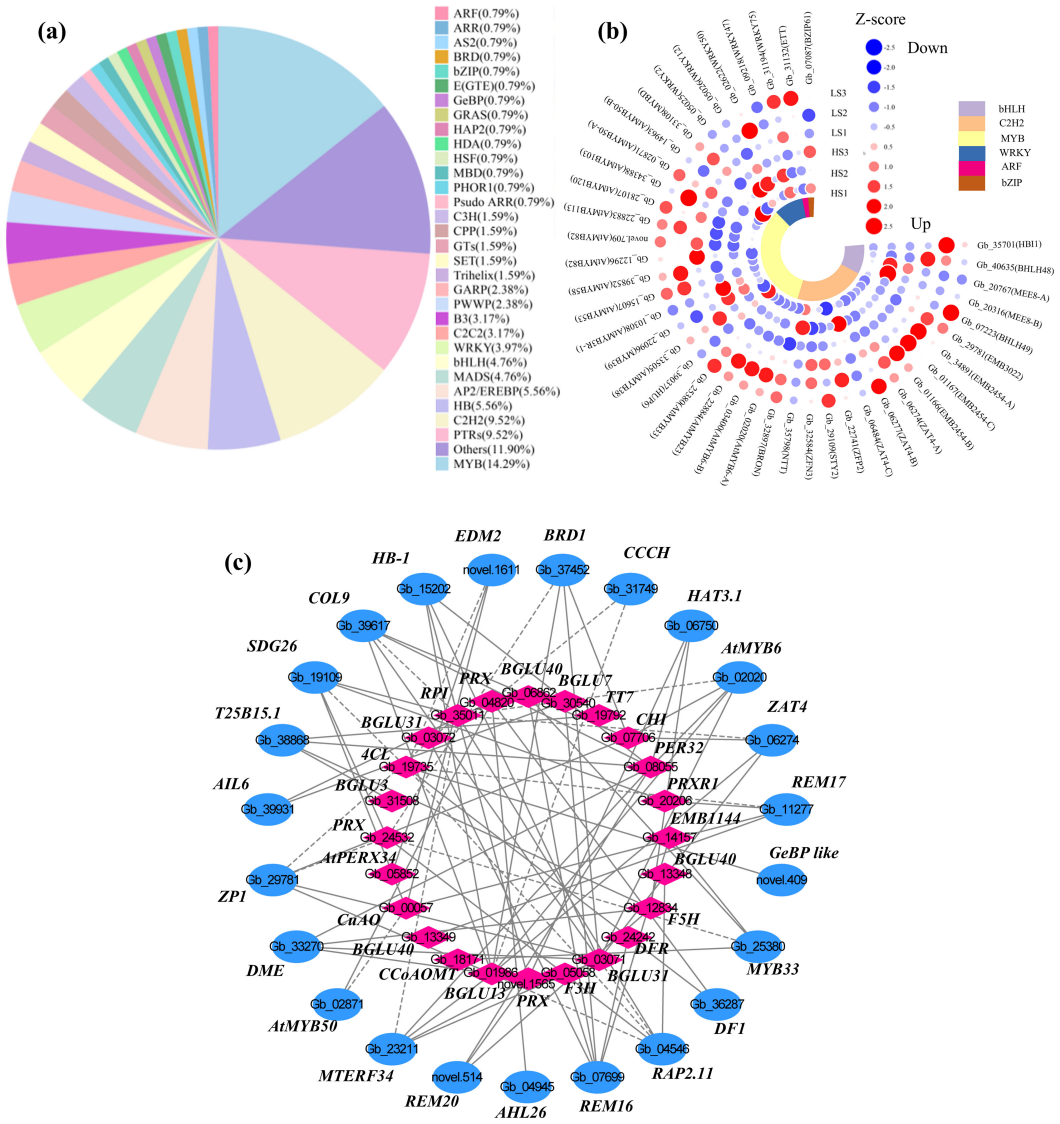
Classification and proportion of differentially expressed transcription factors (TFs) **(a)**, expression profiles of selected TFs **(b)**, and correlation analysis of candidate TFs and differentially expressed genes involved in shikimic acid biosynthesis and downstream metabolism **(c)** in the leaves of *G*. *biloba*. Detailed information about these genes is presented in **Supplementary Table S5**.

A total of 126 differentially expressed TFs were identified in this study (**Supplementary Table S5**). The highest abundance of TF family was MYB (18, 14.29%), followed by PTRs (12, 9.52%), C2H2 (12, 9.52%), HB (7, 5.56%) and AP2-EREBP (7, 5.56%) (**Figure 5a**). Notably, only four *MYBs* were upregulated, while the other 14 *MYBs* were significantly downregulated in HS compared to LS (**Figure 5b**). In addition, most C2H2, bHLH and ARF family genes were downregulated, while *WRKYs* and *bZIPs* were more upregulated in HS compared to LS (**Figure 5b**).

Pearson correlation analysis between the transcript levels of differentially expressed TFs and shikimic acid concentration identified 28 TFs with closely correlations (R ≥ 0.92 or ≤ -0.92) (**Supplementary Table S6**). Among these, nine TFs, such as *enhanced downy mildew 2* (*EDM2*) and *set domain group 26* (*SDG26*), showed a positive correlation with shikimic acid content (**Supplementary Table S6**). The remaining 19 TFs, such as *constans-like 9* (*COL9*) and *myb domain protein 50* (*ATMYB50*), displayed a negative correlation with shikimic acid content (**Supplementary Table S6**).

Correlation analysis was performed to further calculate the correlation coefficients between these 28 TFs and DEGs participating in shikimic acid biosynthesis and downstream metabolism (**Figure 5c**). Each TF was closely correlated with 1–6 DEGs involved in shikimic acid biosynthesis and downstream metabolism (**Figure 5c**; **Supplementary Table S7**). Notably, *related to AP2 11* (*RAP2.11*) and *reproductive meristem 16* (*REM16*) had the most numbers of closely correlated DEGs (**Figure 5c**; **Supplementary Table S7**). *RAP2.11* and *REM16* were considered to be key TFs in regulating shikimic acid biosynthesis and downstream metabolism.

### 3.5 Association analysis of metabolome and transcriptome

**Figure 6:**
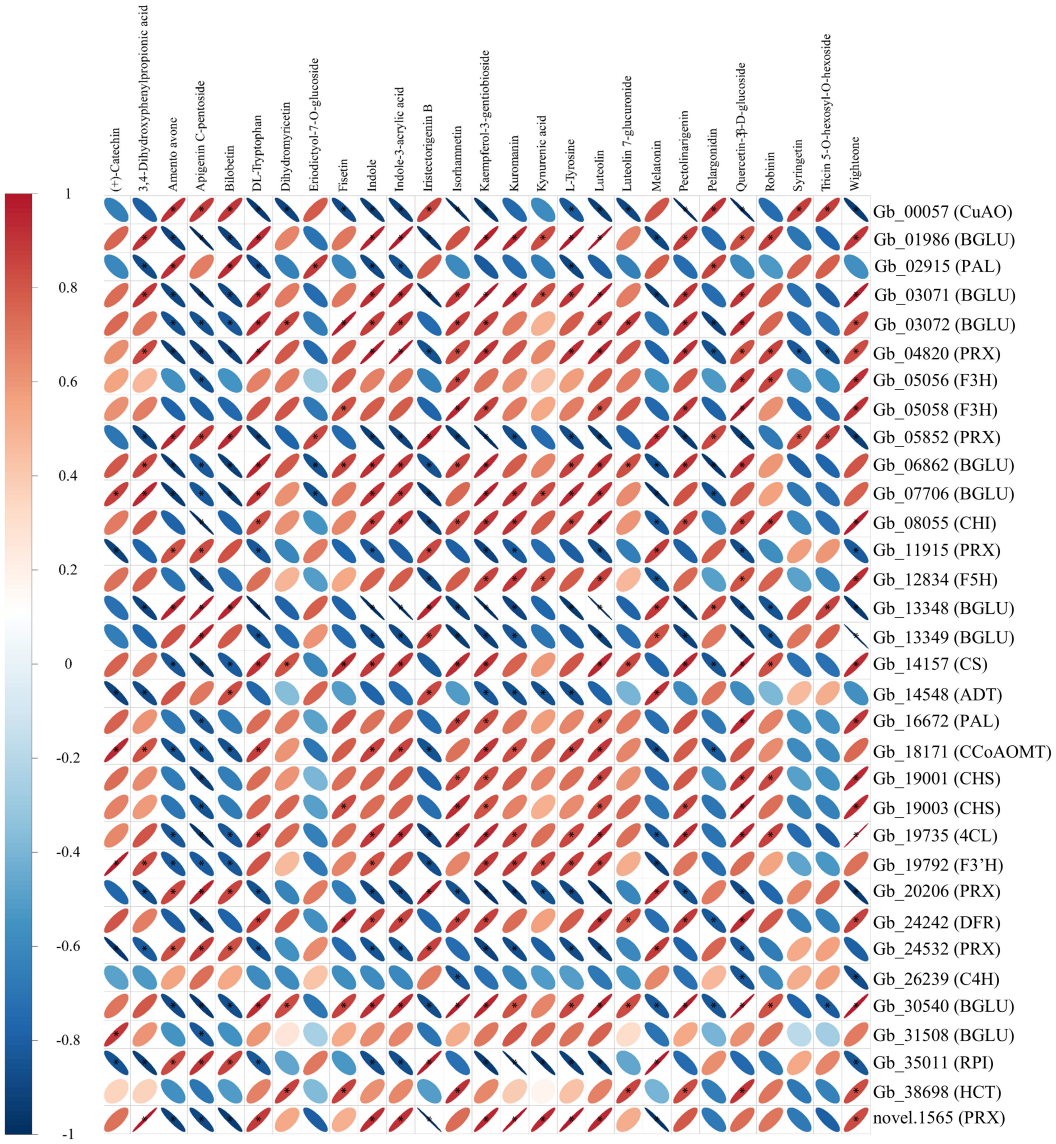
Correlation heatmap of differentially abundant metabolites and differentially expressed genes involved in shikimate biosynthesis and downstream metabolism. ‘*’ indicates a significant correlation (|R| ≥ 0.8, *P*-value < 0.05.) between the metabolites and genes. Detailed information about these genes is provided in **Supplementary Table S5**.

The correlation between DAMs and DEGs that are involved in shikimate biosynthetic and downstream metabolic pathways were analyzed (**Figure 6**). Apigenin c-pentoside, kaempferol-3-gentiobioside and luteolin were three metabolites having the highest numbers of correlated DEGs (**Figure 6**). Specifically, kaempferol-3-gentiobioside, apigenin c-pentoside and luteolin were positively correlated with 19, 8 and 19 DEGs, and were negatively correlated with 10, 20 and 8 DEGs, respectively (**Figure 6**). In addition, *beta glucosidase 7* (*BGLU7*), *Arabidopsis thaliana peroxidase 34* (*AtPERX34*), *peroxidase* (*PRX*) and *copper amine oxidase* (*CuAO*) were found to have the largest numbers (21-22) of correlated DAMs (**Figure 6**). Therefore, the three DAMs and four DEGs may play key roles in the shikimic acid biosynthesis and downstream metabolism in the leaves of *G. biloba*.

## 4 Discussion

### 4.1 The greater synthesis and lower downstream metabolism of shikimic acid are essential for higher shikimic acid concentration in the leaves of *G. biloba*

The biosynthesis and downstream metabolism of shikimic acid determine the final shikimic acid concentration in *G. biloba* leaves. Notably, in the upstream synthesis of shikimic acid, both *MDH* and *RPI* were significantly upregulated in HS compared to LS. Functionally, MDH catalyzes the formation of OAA (as a precursor of PEP), and RPI generates Ru5P, which can be transformed into E4P (Stover et al., 2000). E4P and PEP constitute the essential substrate pairs for shikimic acid biosynthesis (Kruger and von Schaewen, 2003). These findings suggest that the upregulation of *MDH* and *RPI* play key roles in higher shikimic acid concentration in HS *vs*. LS.

Previous studies have demonstrated that the attenuated downstream metabolic flux exhibit significant accumulation of shikimic acid in plants (Bochkov et al., 2011; Eloy et al., 2017). The biosynthesis of aromatic amino acids and flavonoids are two vital metabolic pathways of downstream shikimic acid metabolism. Correspondingly, the abundances of several aromatic amino acids and flavonoid-related metabolites, such as phenylalanine, tryptophan, tyrosine, dihydromyricetin, (+)-catechin and luteolin, were significantly lower in HS *vs*. LS. Based on the association analysis, luteolin was identified as a key metabolite. In this study, the expression levels of multiple genes, such as *CS*, *CHS*, *DFR*, *PRX*, *ATPERX34*, *BGLU7* and *CuAO*, which are involved in biosynthesis of aromatic amino acids and flavonoids were markedly downregulated in HS compared to LS. Additionally, Integrated analysis of metabolomic and transcriptomic data revealed that *PRX*, *ATPERX34*, *BGLU7*, and *CuAO* may play pivotal roles in shikimate biosynthesis and downstream metabolism. In maize (*Zea mays*), suppression of *CHS* results in lower flavonoid production and higher accumulation of shikimic acid derivatives (Eloy et al., 2017). Similarly, inhibition of the *CS* in *Petunia hybrida* leads to reduced flavonoid accumulation (Zhong et al., 2020). These results suggest that the downregulation of metabolites and genes participating in downstream metabolism of shikimic acid contribute to higher concentration of shikimic acid in HS than that in LS.

### 4.2 Changes in expression levels of genes involved in phytohormone metabolism might contribute to higher shikimic acid accumulation

Previous studies have shown that ABA and IAA could affect the metabolism of flavonoid (Jeong et al., 2004; Dong and Lin, 2021), probably regulating the shikimic acid accumulation in *G. biloba* leaves. For instance, exogenous application of ABA increases the levels of flavonoid-related metabolites, such as isorhamnetin-3-O-gallate and dihydromyricetin, in *Rhododendron chrysanthum* after UV-B radiation (Yu et al., 2024). ABA positively regulates flavonoid accumulation by upregulating *F3’H* under drought stress in *G. biloba* (Yu et al., 2022). Auxin is also reported to be positively correlated with flavonoid accumulation by upregulating *CHS* in Arabidopsis root galls (PÄSold et al., 2010). In this study, the abundances of ABA and IAA, and the mRNA levels of *ABF*, *GER*, *BIG*, *CAND1*, *LAX3* and *GH3.1*, which are involved in ABA and IAA signaling and metabolism, were significantly downregulated in HS compared to LS. These results indicate that the downregulation of ABA and IAA, along with the lower expression of genes participating in ABA and IAA signaling and metabolism, play vital roles in limiting flavonoid biosynthesis, thus contributing to higher shikimic acid accumulation in HS than that in LS.

### 4.3 Transcription factors play an important role in regulating shikimic acid accumulation

In this study, the most abundant TFs belonged to the MYB family members (**Figure 5a**). Furthermore, in the co-expression network (**Figure 5c**), four TFs—*MYB6*, *ZP1*, *RAP2.11*, and *MTERF34*—exhibited the most connections with key structural genes, suggesting their potential regulatory roles in regulating shikimic acid biosynthesis and downstream metabolism. Previous studies have revealed that *MYBs*, *C2H2s* and *ERFs* play key roles in regulating flavonoid biosynthesis (Li et al., 2024a), probably affecting the shikimic acid concentration in *G. biloba* leaves. For instance, overexpression of *MYB6* brings about upregulation of *DFR2*, resulting in larger concentrations of anthocyanin and proanthocyanidins in poplar (Wang et al., 2019). As a C2H2 family member, *ZAT* could regulate flavonoid biosynthesis by interacting with the promoter regions of genes involved in flavonoid biosynthesis, such as *CHS* in *Macadamia integrifolia* under high temperature stress (Yang et al., 2023). AP2/ERF transcription factors can directly target the key genes in the flavonoid biosynthesis pathway, thereby regulating the synthesis of flavonoid in *Solanum lycopersicum* and *Citrus* (Zhao et al., 2020; Cao et al., 2024). These results suggest that TFs play key roles in regulating shikimic acid downstream metabolism, thus bringing about higher shikimic acid accumulation in HS than that in LS.

## 5 Conclusion

Taken together, compared to LS, HS exhibited significantly higher concentration of shikimic acid but lower levels of downstream aromatic amino acids and flavonoids. Correspondingly, a number of metabolites and genes that are related to biosynthesis and downstream metabolic partitioning of shikimic acid were significantly differentially regulated. For instance, the mRNA levels of *MDH* and *RPI*, that are involved in shikimic acid biosynthesis, were higher in HS *vs*. LS. The abundances of luteolin and dihydromyricetin and the mRNA levels of *CHS* and *F3H*, that are implicated in downstream metabolism of shikimic acid, were lower in HS *vs*. LS. The abundances of ABA and IAA in HS were lower than those in LS. Moreover, 28 transcription factors, such as *ERFs*, *C2H2*s and *MYBs* that play roles in accumulation of shikimic acid were identified. These results suggest that metabolites and structural genes involved in biosynthesis and downstream metabolism of shikimic acids, and ABA, IAA and transcript factors play key roles in shikimic acid accumulation in *G. biloba* leaves.

## Data Availability

The datasets presented in this study can be found in online repositories. The names of the repository/repositories and accession number(s) can be found in the article/**Supplementary Material**. The sequencing data have been deposited in GSA and are publicly available under accession number CRA025492.

## Supplementary material

Supplementary Table 1 Primers used for RT-qPCR.

Supplementary Table 2 Identified metabolites in *G. biloba* leaves.

Supplementary Table 3 The significantly differentially abundant metabolites (DAMs).

Supplementary Table 4 Summary of RNA-sequencing reads.

Supplementary Table 5 The significantly differentially expressed genes (DEGs).

Supplementary Table 6 Candidate TFs involved in shikimic acid biosynthesis and metabolism.

Supplementary Table 7 Correlation analysis between TFs and structural genes.

Supplementary Figure 1 Volcano plots and KEGG enrichment analysis of DAMs.

Supplementary Figure 2 The validation of DEGs by RT-qPCR.

Supplementary Figure 3 DEGs involved in abscisic acid and auxin metabolism.

Supplementary Figure 4 Simplified shikimic acid biosynthetic and downstream metabolic pathways.
