# Supplementary material for: Metabolomic and transcriptomic analyses unveil the accumulation of shikimic acid in the leaves of Ginkgo biloba
Source: Front Plant Sci. 2025 Aug 22;16:1631197. doi: 10.3389/fpls.2025.1631197 (PMC12411440; doi:10.3389/fpls.2025.1631197)
Supplement: Supplementary Figure 1 — Volcano plots and KEGG enrichment analysis of DAMs. [file DataSheet1.docx]

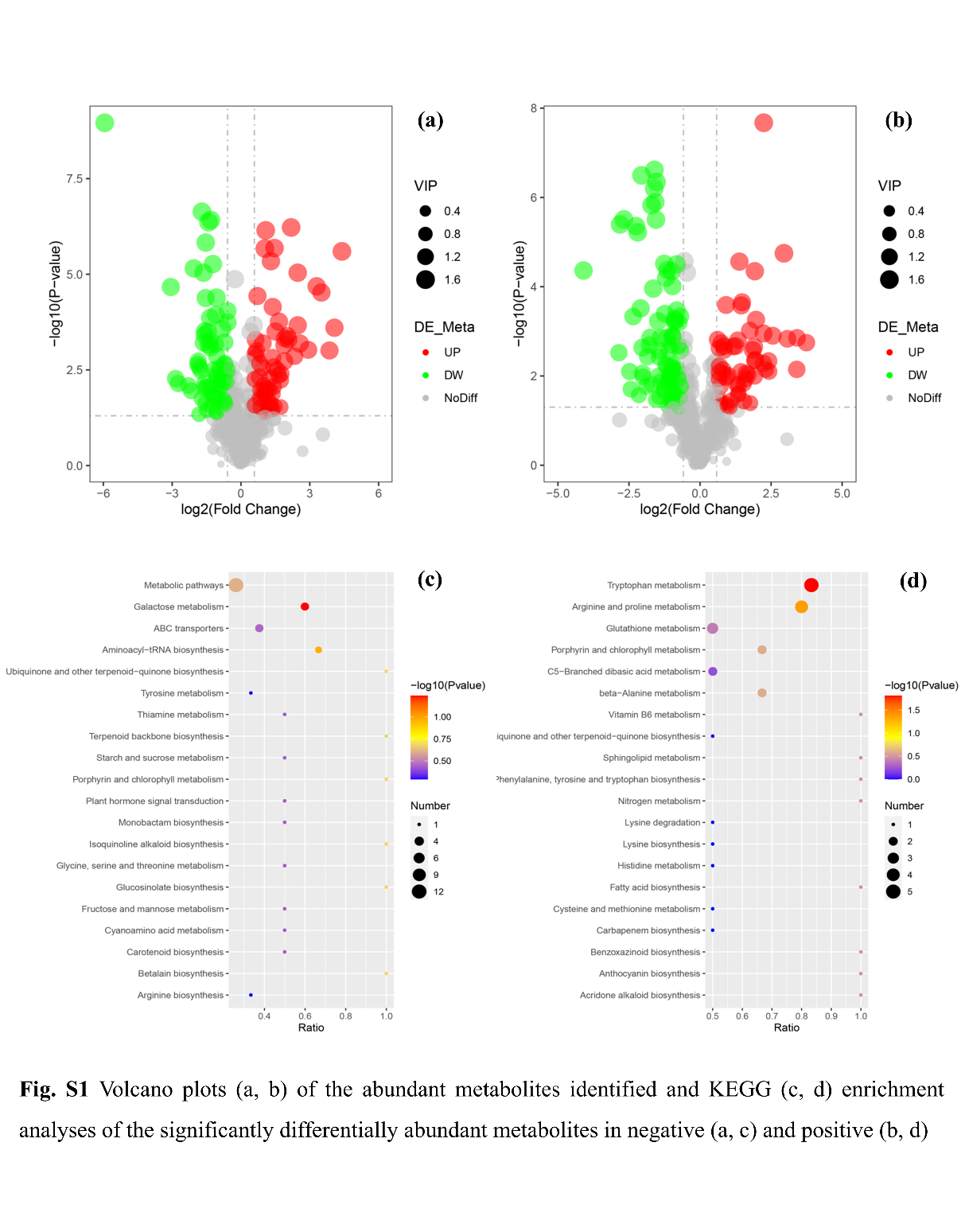


**Fig. S1** Volcano plots (a, b) and KEGG (c, d) enrichment analysis of the significantly differentially abundant metabolites identified in negative (a, c) and positive (b, d) modes.


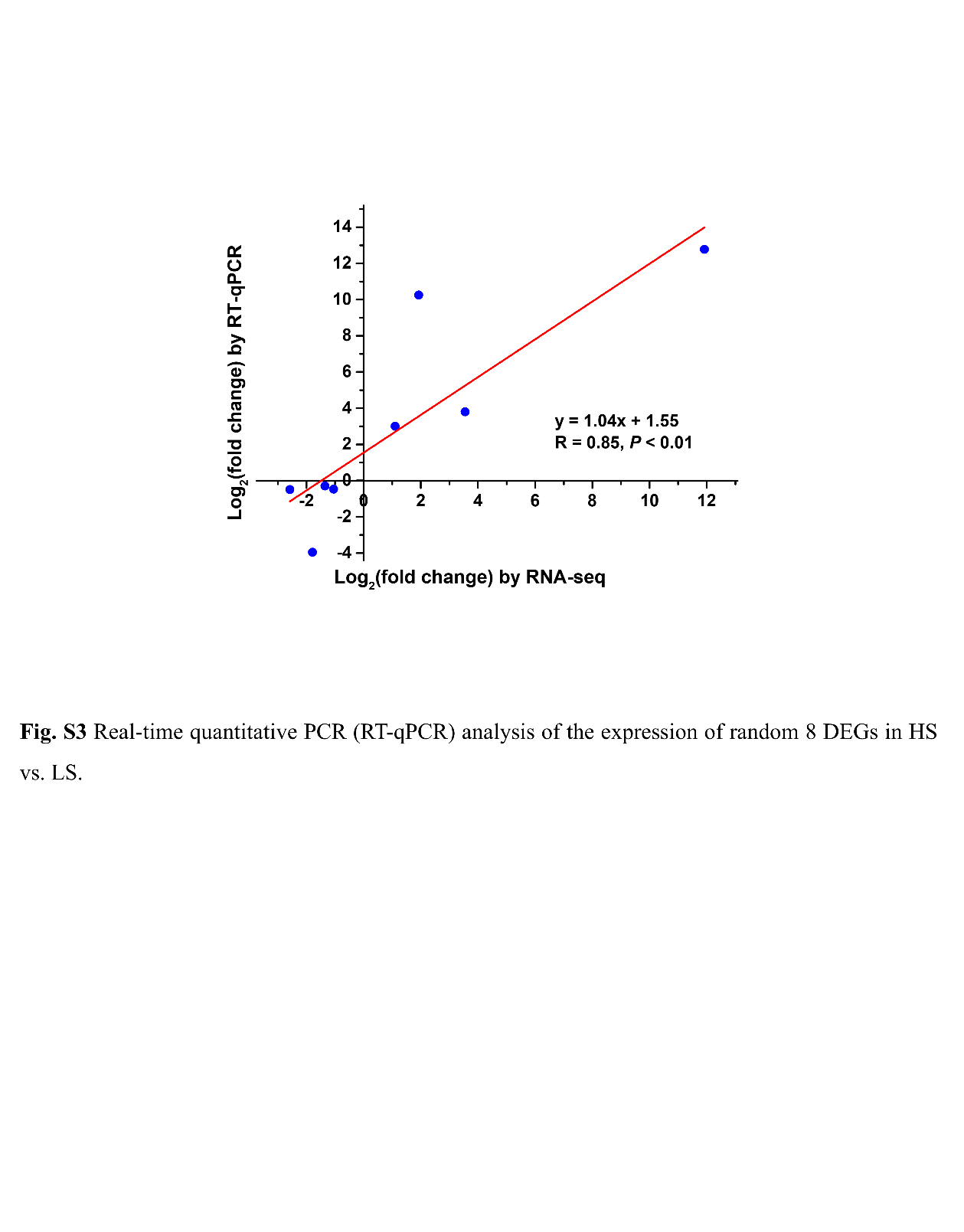


**Fig. S2** The validation of significantly differentially expressed genes in HS vs. LS by real-time quantitative PCR (RT-qPCR).


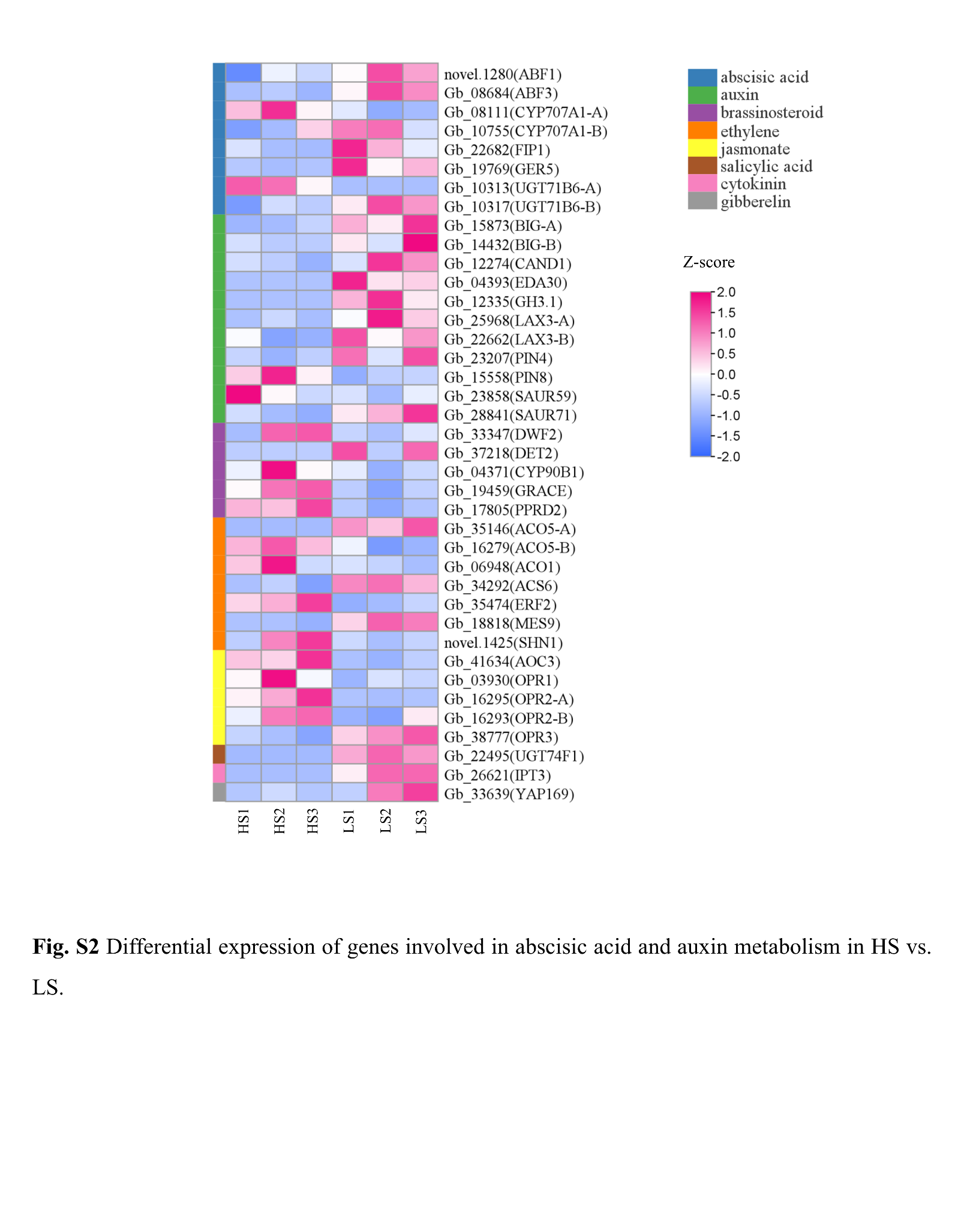


**Fig. S3** Differentially expressed genes involved in phytohormone metabolism in HS vs. LS.


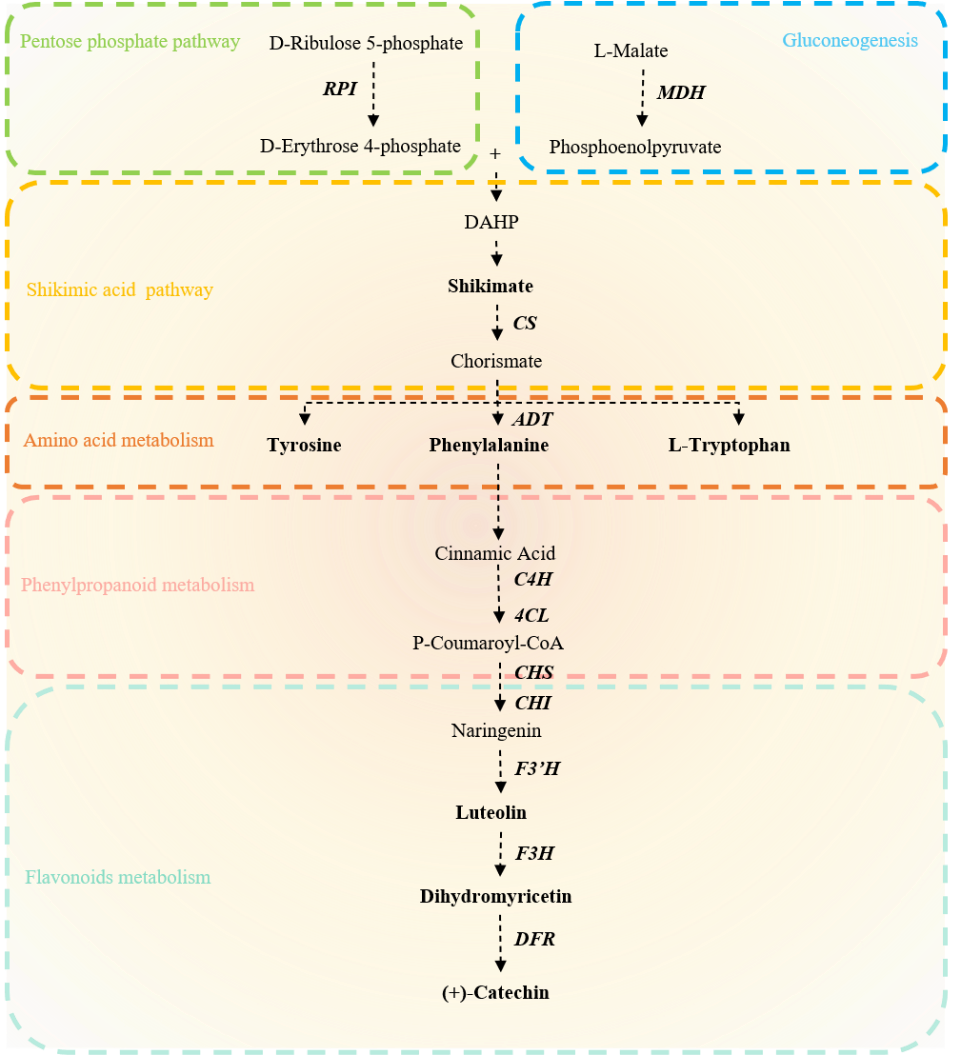


**Fig**. **S4** Simplified shikimic acid biosynthesis and downstream metabolism in HS compared to LS. Pathways are color coded. Dashed arrows represent multi-step reactions. DAMs and DEGs are in black bold fonts. The detailed shikimic acid biosynthesis and downstream metabolism pathway is shown in Figure 4.
